# Expression Pattern of Kv11 (Ether à-go-go-Related Gene; erg) K^+^ Channels in the Mouse Retina

**DOI:** 10.1371/journal.pone.0029490

**Published:** 2011-12-19

**Authors:** Sönke Cordeiro, Daria Guseva, Iris Wulfsen, Christiane K. Bauer

**Affiliations:** 1 Institut für Neurophysiologie, Medizinische Hochschule Hannover, Hannover, Germany; 2 Physiologisches Institut, Universität zu Kiel, Kiel, Germany; 3 Institut für Pharmakologie für Pharmazeuten, Universitätsklinikum Hamburg-Eppendorf, Hamburg, Germany; 4 Institut für Zelluläre und Integrative Physiologie, Universitätsklinikum Hamburg-Eppendorf, Hamburg, Germany; Indiana University School of Medicine, United States of America

## Abstract

In response to light, most retinal neurons exhibit gradual changes in membrane potential. Therefore K^+^ channels that mediate threshold currents are well-suited for the fine-tuning of signal transduction. In the present study we demonstrate the expression of the different Kv11 (ether-à-go-go related gene; erg) channel subunits in the human and mouse retina by RT PCR and quantitative PCR, respectively. Immunofluorescence analysis with cryosections of mouse retinae revealed the following local distribution of the three Kv11 subunits: Kv11.1 (m-erg1) displayed the most abundant expression with the strongest immunoreactivity in rod bipolar cells. In addition, immunoreactivity was found in the inner part of the outer plexiform layer (OPL), in the inner plexiform layer (IPL) and in the inner segments of photoreceptors. Immunoreactivity for Kv11.2 (m-erg2) was observed in the outer part of the OPL and throughout the IPL. Double-labeling for vGluT1 or synaptophysin indicated a mainly presynaptic localization of Kv11.2. While no significant staining for Kv11.3 (m-erg3) was detected in the neuronal retina, strong Kv11.3 immunoreactivity was present in the apical membrane of the retinal pigment epithelium. The different expression levels were confirmed by real-time PCR showing almost equal levels of Kv11.1 and Kv11.2, while Kv11.3 mRNA expression was significantly lower. The two main splice variants of Kv11.1, isoforms a and b were detected in comparable levels suggesting a possible formation of cGMP/cGK-sensitive Kv11.1 channels in photoreceptors and rod bipolar cells. Taken together, the immunohistological results revealed different expression patterns of the three Kv11 channels in the mouse retina supposing distinct physiological roles.

## Introduction

The vertebrate retina is a neuronal network which consists of six major cell types. The incoming light is detected by photoreceptors and the information is subsequently passed through bipolar cells to ganglion cells which form the optic nerve. The processing of the incoming signals already occurs in the retina via feedback mechanisms and lateral connections between retinal neurons [1]–[3]. In contrast to some subtypes of amacrine cells and the ganglion cells which are able to generate action potentials, neurotransmitter release from most retinal neurons is triggered by only gradual changes of the membrane potential. Therefore, a fine tuning of the membrane potential is of particular importance in these retinal neurons.

Kv11 (ether à-go-go related gene; erg) K^+^ channels belong to the EAG family of voltage-gated K^+^ channels. Most members of this family (Kv10 or eag, Kv11 or erg, and Kv12 or elk channels) share an activation threshold at relatively negative potentials [4]. Accordingly, these channels are able to control the membrane potential in retinal neurons in their rested and activated state. An involvement of Kv10.1 (eag1) channels in I_Kx_, the counter-current to the dark-current in photoreceptors, has been suggested [5], and more recently, the presence of Kv10.1 and Kv10.2 (eag2) channels in most layers of the rat retina was demonstrated [6]. When the two members of the Kv11 channels Kv11.2 and Kv11.3 were first cloned from the rat nervous system, a mRNA expression of all three members of this K^+^ channel family in the rat retina was detected, too [7]. In situ hybridization experiments with mouse embryos confirmed the mRNA expression of the three Kv11 channel subunits in the neural layer of the retina [8]. However, beside a possible involvement of Kv11.1-mediated currents in shaping the dark response in horizontal cells [9], nothing is known about the expression pattern nor the physiological role of Kv11 channels in the retina.

In the present study we unveiled the expression patterns of all three Kv11 subunits in the mouse retina. Our results point to different physiological roles for the different subunits.

## Materials and Methods

### Ethics statement

All animal experiments were approved by the local authorities of the City of Hannover (Niedersächsisches Landesamt für Verbraucherschutz und Lebensmittelsicherheit) and were conducted in accordance with the German law on the protection of experimental animals and the European Communities Council Directive of November 24 1986 (86/609/EEC).

For the use of human material, tenets of the Declaration of Helsinki were followed, informed consent was obtained, and Institutional Human Experimentation Committee approval was granted for the studies.

### RT PCR experiments

We investigated the mRNA expression of all three Kv11 channel subunits in mouse and human retinas by RT PCR. To yield mRNA from mouse retinas, C57Bl/6J mice were sacrificed by cervical dislocation and the retinas were isolated immediately from the enucleated eye cups and put into lysis buffer from RNeasy Mini Kit for RNA extraction (Qiagen). Human retinal mRNA was taken from human donor eyes within 24 hours after death. The RNA was reverse transcribed and used for RT PCR reactions as described elsewhere [10]. The oligonucleotides (each pair spanning at least 1 intron) listed in table 1 were used for specific amplification.

**Table 1:** List of oligonucleotides used for specific amplification in this study.

gene	organism (Acc. No.)	Sequence	first base
Kv11.1 (erg1)	human (NM_000238)	fd CTTCAAGGCCGTGTGGGACT	1616
		rev CAGGTTGTGCAGCCAGCCGA	2168
Kv11.2 (erg2)	human (NM_173092)	fd GATGAACAGGCTGGAGTCCC	2309
		rev GTGGCCCCAACTCCCTGC	2795
Kv11.3 (erg3)	human (NM_033272)	fd GGAACTGCCAGGTACCACAT	2243
		rev GTTAGAAAGTGATCAGAAAA	2799
Kv11.1 (m-erg1a)	mouse (NM_013569)	fd CTCATGACACCAACCACAGG	746
		rev GTTGTCCATGGCAGAAACCT	981
Kv11.1 (m-erg1b)	mouse (AF012869)	fd TAAGTCCTCCATGGCGATTC	258
		rev TTGAAGGGGCTGTAGTGGAG	467
Kv11.2 (m-erg2)	mouse (NM_001037712)	fd GCCAATCAGGTGCTGCCCCT	2225
		rev GAGGTCTGACGACACGCGGG	2418
Kv11.3 (m-erg3)	mouse (NM_133207)	fd CAACCAGACTCCATGGTGAA	3092
		rev GGCAGCTCTCTGAAGTCCTG	3255
Ribosomal protein L32	mouse (NM_172086)	fd CGGGATGGAGCTGGAGGTGCTGCTGA	300
		rev GGAATTCGCCAGCTGTGCTGCTCTTTC	407

### Real-time PCR experiments

For comparative quantification of the expression of the three Kv11 channel subunits we extracted RNAs from retinas of C57Bl/6J mice (2 male, 2 female, 1 month old) using the RNeasy Mini Kit (Qiagen). The mRNAs were reverse transcribed and the real-time experiments performed as described earlier [11]. The expression levels of Kv11 subunits were quantified relative to the expression of ribosomal protein L32. The initial ratios of Kv11 to L32 were calculated using the equation 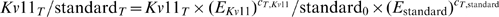 (*X*_T_  =  threshold number of molecules; *X*_0_  =  initial number of molecules; *C*_T_  =  threshold cycle of amplification; *E*  =  efficiency of amplification). The efficiencies were calculated with standard curves using retina cDNA (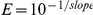). The oligonucleotide primers used for real-time PCR are listed in table 1.

### Immunohistochemistry

To access the retinal distribution patterns of the Kv11 channel subunits, we performed immunohistological experiments with eyes from C57Bl/6J mice. For these studies 1-month old animals (n = 10) of both sexes were used. Prior to enucleation, the mice were anesthetized by intraperitoneal injection of 16% sodium pentobarbital solution (Narcoren®, Merial, Hallbergmoos, Germany, 5 µl/g body weight) and transcardially perfused with 4% formaldehyde in 0.1 M sodium cacodylate buffer, pH 7.3. After enucleation the eyes were post-fixed in 4% paraformaldehyde for 30 minutes and cryoprotected in graded sucrose solutions (10%, 20% and 30%) in 0.1 M cacodylate buffer, pH 7.3. The 14 µm cryosections were permeabilized in blocking buffer (PBS with 0.2% Triton X-100, 0.02% sodium azide, 5% donkey serum). The primary antibodies listed in table 2 were applied overnight at 4°C.

**Table 2:** List of primary antibodies used in this study.

antigen	antiserum	source	Dilution
erg1	rabbit anti-erg1	Chemicon AB5930	1∶1000
erg2	rabbit anti-erg2	this report	1∶1000
erg3	rabbit anti-erg3	this report	1∶500
PKCα	mouse anti-PKCα	Santa Cruz Biotechnology sc-8393	1∶250
calbindin	mouse anti-calbindin	Abcam ab9481	1∶1000
vGluT1	guinea pig anti-vGluT1	Synaptic Systems 135304	1∶5000
ChAT	goat anti-choline acetyltransferase	Chemicon (Millipore) AB144P	1∶100
synaptophysin	goat anti-synaptophysin	Santa Cruz Biotechnology sc-7568	1∶50
GAD2/GAD65	guinea pig anti-GAD2/GAD65	Synaptic Systems 198104	1∶1000
ezrin	mouse anti-ezrin	Abcam ab4069	1∶250

The immunoreactivities were visualized by appropriate secondary antibodies: donkey anti-mouse conjugated to DyLight 488, donkey anti-rabbit DyLight 649,donkey anti-goat DyLight 549 or donkey anti-guinea pig DyLight 488 (Jackson ImmunoResearch). The cell nuclei were counter stained by 10 minutes incubation with bis-benzimide solution (Hoechst 33258, Sigma). Confocal imaging was performed with a TCS SP2 AOBS scan head and an inverted Leica DM IRB.

### Antibody production

For the generation of anti-Kv11.2 and anti-Kv11.3 antibodies, fusion proteins of glutathione-S-transferase and rat Kv11.2 (amino acids 722–950; Acc No. NP_446389) or rat Kv11.3 (amino acids 976–1090; Acc No. NP_571987) were prepared by subcloning the corresponding cDNA fragments into the pGEX vector (Pharmacia) and expression in E. coli. The fusion proteins were purified with glutathione sepharose 4B beads (Pharmacia) and rabbits were immunized with the fusion proteins.

Prior to the immunohistological experiments, suitability of the used Kv11 antibodies was tested by immunocytochemistry on HEK293 cells transiently transfected with rat Kv11.1, Kv11.2 or Kv11.3 cDNA. We used rat cDNA because the antibodies were also raised against parts of the rat Kv11 subunits. To allow a direct comparison of subcellular Kv11 subunit location with antibody staining we transfected Kv11 channel subunits with a C-terminal EGFP tag subcloned from the pEGFP-N vector (Clontech). Goat anti-rabbit antibodies conjugated to Alexa Fluor 546 (Molecular Probes; 1∶4000) were used to visualize Kv11 channel immunoreactivity. The three Kv11 antibodies were found to label specifically transfected cells and the Kv11 immunoreactivity exhibited identical subcellular distribution compared to the EGFP signal of the tagged Kv11 channels (Figure S1). Weak cross-reactivity could be detected by staining Kv11.2-transfected cells with the anti-Kv11.1 antibody as well as the Kv11.1-transfected cells with the anti-Kv11.2 antibody. Nevertheless, the antibodies are still suitable for specific staining because of the much stronger affinity to their specific targets. This specificity is also confirmed by the very different fluorescent signals in immunohistological stainings of the retina (see Results).

## Results

**Figure 1:**
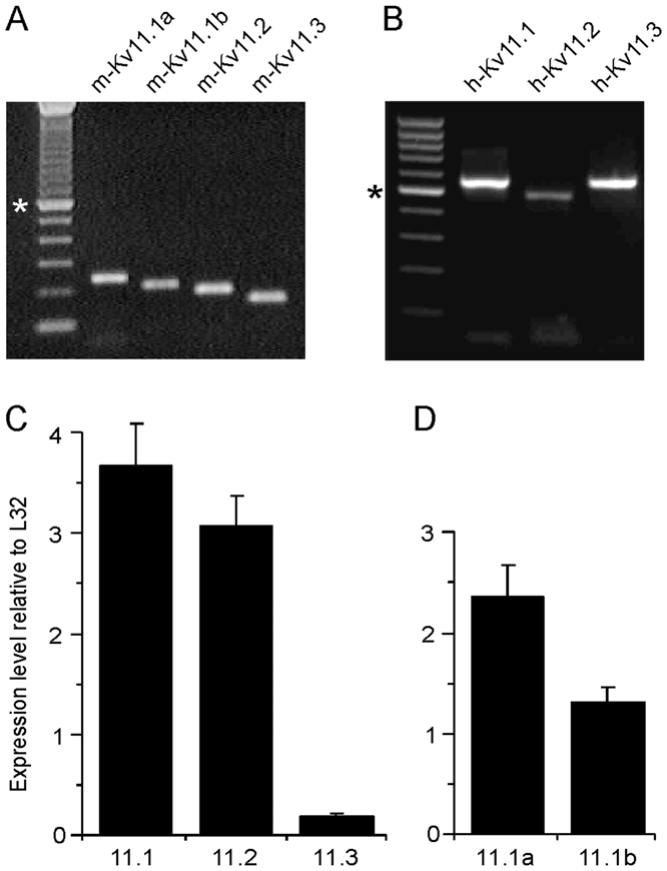
mRNA expression of Kv11 channel subunits in the mouse and human retina. RT PCR experiments showing the expression of transcripts of all three Kv11 channel subunits in the mouse (A) and human (B) retina; * indicates the 600 (A) or 500 bp (B) fragments of the molecular weight marker (100 bp ladder). In A the expression of the most abundant splice-variants of Kv11.1, isoforms a and b, in the mouse retina is also shown. (C) Real-time PCR experiments for the three Kv11 channel genes revealed comparable levels of expression for Kv11.1 and Kv11.2, while Kv11.3 is expressed at a significantly lower level in the mouse retina. (D) Using primers specific for the two most abundant splice-variants of Kv11.1 we detected the two splice-variants in a ratio of 1.8∶1. The expression levels of erg genes were normalized to the expression level of the ribosomal protein L32. Each experiment was carried out with four retinal mRNA preparations.

Previous studies demonstrated the expression of the three Kv11 channel subunits only in rat retinas [7]. We first analyzed Kv11 channel mRNA expression in human and mouse retinas. By RT PCR experiments we confirmed the expression of all three subunits in the mouse as well as in the human retina (Fig. 1A and B, respectively). By real-time PCR experiments we were able to show that Kv11.1 and Kv11.2 are expressed in a comparable level in the mouse retina while Kv11.3 expression was significantly lower (Fig. 1C). This is in agreement with Shi *et al*. [7] who also detected only very low levels of Kv11.3 expression in the retina. In the CNS Kv11.1 channels are mainly composed of two splice variants, Kv11.1 isoform a (erg1a) and Kv11.1 isoform b (erg1b), which differ significantly in their biophysical properties and in their modulation by cGMP [11], [12]. We performed real-time PCR experiments to demonstrate their mRNA expression in the retina. As seen in Fig. 1D, both splice variants were detected in relatively high levels with a ratio of 1.81∶1 for isoform a:isoform b. These results point to a possible formation of heteromeric cGMP-sensitive Kv11.1a/b channels in the retina.

**Figure 2:**
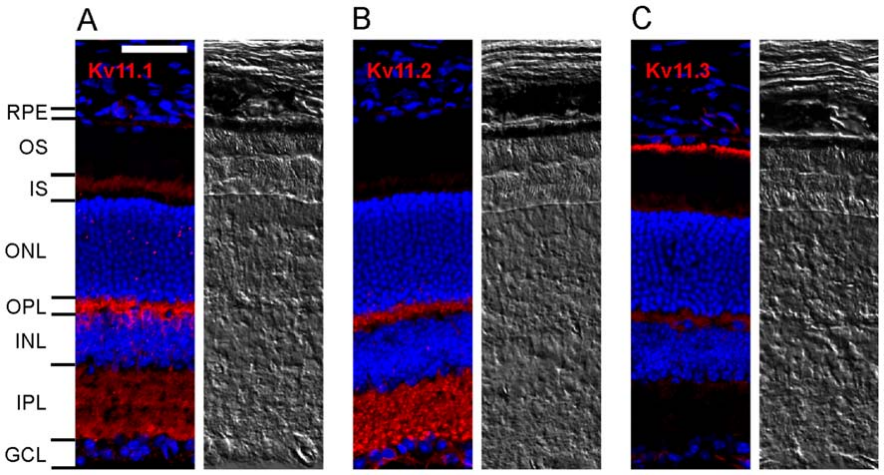
Distribution of Kv11.1, Kv11.2 and Kv11.3 in the mouse retina. Confocal fluorescence micrographs of mouse retinas labeled with antibodies against Kv11.1, Kv11.2 and Kv11.3 and counter stained for cell nuclei. (A) The Kv11.1 immunoreactivity was observed in IS, the OPL, the INL and the IPL. (B) Immunoreactivity for Kv11.2 was detected in the OPL and in the IPL. (C) Immunostaining of retina with the Kv11.3-specific antibody revealed no significant signals in the neuronal retina, but in the retinal pigment epithelium. GCL: ganglion cell layer; IPL: inner plexiform layer; INL: inner nuclear layer; OPL: outer plexiform layer; ONL: outer nuclear layer; IS: photoreceptor inner segments; OS: photoreceptor outer segments; RPE: retinal pigment epithelium.

The distribution of Kv11 channel subunits in the mouse retina was investigated using the specific Kv11.1, Kv11.2 and Kv11.3 antibodies in immunofluorescence experiments on eye cup slices. These experiments demonstrated strong expression of both, Kv11.1 and Kv11.2 subunits, in the neuronal retina while there was only a very small fraction of Kv11.3-positive cells in the neuronal retina (Fig. 2). Kv11.1 subunits displayed the most abundant expression. The immunostaining for Kv11.1 was most prominent in the inner part of the outer plexiform layer (OPL) and in the outer part of the inner nuclear layer (INL), which contains the somata of horizontal cells and bipolar cells. In addition, immunoreactivity was found in the inner plexiform layer (IPL) and in the inner segments of the photoreceptors (IS).

Immunoreactivity for Kv11.2 was present in the outer part of the OPL and throughout the IPL. While Kv11.1 immunoreactivity was found in both, neuronal somata and cell processes, the expression of Kv11.2 subunits was restricted to processes of different retinal neurons. Thus, Kv11.1 and Kv11.2 subunits displayed a different expression pattern within the IPL and the OPL. Nevertheless, a partial overlap in the IPL could point to the presence of heteromeric Kv11 channels, since different Kv11 subunits can assemble to form heteromeric channels [13].

Kv11.3 immunoreactivity was not found at a significant level in the neuronal retina, but we detected strong expression of Kv11.3 subunits in the retinal pigment epithelium.

**Figure 3:**
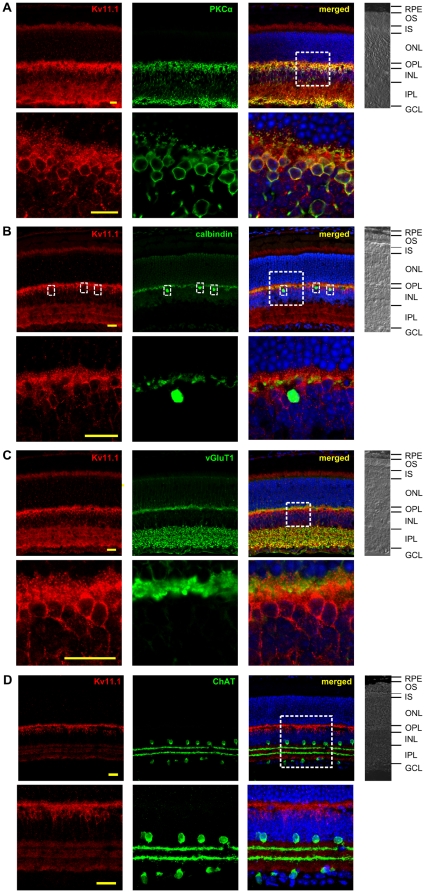
Double-labeling experiments for the identification of Kv11.1 channel subunit expressing cells in the mouse retina. (A) PKCα-specific antibodies were used for double-labeling experiments with Kv11.1. An almost perfect co-localization of both proteins was detected in rod bipolar cells. The box highlights the section shown in the bottom row in higher magnification illustrating the co-localization of Kv11.1 and PKCα. (B) Calbindin-specific antibodies were used for double-labeling experiments with Kv11.1. No significant co-localization of proteins was found in the mouse retina (the small boxes highlight cell bodies of horizontal cells immunolabeled by calbindin). The large box highlights the section shown in the bottom row in higher magnification illustrating the distinct expression of Kv11.1 and calbindin in the mouse retina. (C) Double-labeling experiments with Kv11.1 and vGluT1-specific antibodies. Some co-localization was detected in the IPL, especially in the axonal terminals of bipolar cells. In the OPL no co-localization could be detected. The box highlights the section shown in the bottom row in higher magnification illustrating the distinct expression of Kv11.1 and vGluT1 in the OPL of the mouse retina. (D) Double-labeling experiments with Kv11.1 and ChAT-specific antibodies. The merged data show no co-localization of both proteins. The box highlights the section shown in the bottom row in higher magnification illustrating the distinct expression of Kv11.1 and ChAT. On the right of each panel a part of the bright field picture is shown. RPE: retinal pigment epithelium; OS: outer segments; IS: inner segments; ONL: outer nuclear layer; OPL: outer plexiform layer; INL: inner nuclear layer; IPL: inner plexiform layer; GCL: ganglion cell layer. Scale bars: 20 µm. The results of counter staining of cell nuclei (blue) is included in the merged pictures shown on the right.

Double-labeling experiments were performed in the attempt to further circumscribe the Kv11 immunoreactivity to certain neurons or cell layers. The only study on the localization of Kv11 channel proteins in the retina so far reported an expression of Kv11.1 subunits in somata and primary dendrites of horizontal cells [9]. Therefore, we used the Kv11.1 antibody in combination with the horizontal cell marker calbindin [14]. Both antibodies stained cell somata in the outer part of the INL. Surprisingly, all cells positive for calbindin were not co-stained with Kv11.1 antibodies (Fig. 3B). As the immunostaining for Kv11.1 in the INL was restricted to the somata in the outer part of this layer, we performed double-labeling experiments for Kv11.1 and the rod bipolar marker PKCα. As seen in Fig. 3A these experiments revealed an almost perfect overlap in the expression of Kv11.1 subunits and PKCα in rod bipolar cells. The overlap is most obvious in the soma membrane and in the axon terminals of these cells in the IPL, but it can also be seen in the rod bipolar dendrites in the inner part of the OPL.

**Figure 4:**
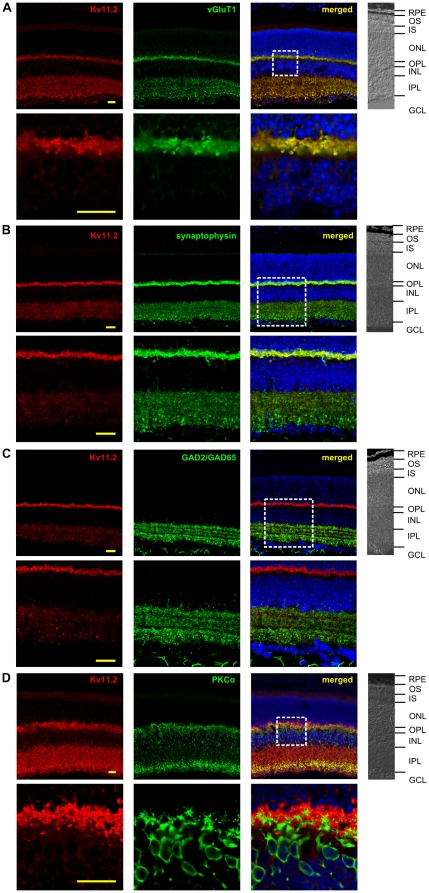
Double-labeling experiments for the identification of Kv11.2 channel subunit expressing cells in the mouse retina. (A) The presynaptic marker of glutamatergic synapses vGluT1 was used for double-labeling experiments with Kv11.2. The merged data show a high degree of co-localization of both proteins. The box highlights the section shown in the bottom row in higher magnification illustrating the co-expression of Kv11.2 and vGluT1 in the IPL and OPL. (B) Double-labeling for the unspecific presynaptic marker synaptophysin and for Kv11.2. The merged data show a high degree of overlap in the OPL. In the IPL, Kv11.2 immunoreactivity is present in a subset of synaptophysin-positive presynaptic structures. The box highlights the section shown in the bottom row in higher magnification. (C) Double-labeling for the GABAergic marker GAD2/GAD65 and for Kv11.2. The merged data show no co-localization of both proteins. The box highlights the section shown in the bottom row in higher magnification illustrating the distinct expression of Kv11.2 and GAD2/GAD65. (D) A PKCα-specific antibody was used for double-labeling experiments with the Kv11.2-specific antibody. Some co-localization was detected in the IPL, especially in the axonal terminals of bipolar cells. In the OPL no co-localization could be detected. The box highlights the section shown in the bottom row in higher magnification illustrating the distinct expression pattern of Kv11.2 and PKCα in the OPL. On the right of each panel a part of the bright field picture is shown. RPE: retinal pigment epithelium; OS: outer segments; IS: inner segments; ONL: outer nuclear layer; OPL: outer plexiform layer; INL: inner nuclear layer; IPL: inner plexiform layer; GCL: ganglion cell layer. Scale bars: 20 µm. The results of counter staining of cell nuclei (blue) is included in the merged pictures shown on the right.

In contrast to this postsynaptic distribution of Kv11.1 subunits in the OPL, Kv11.2 seemed to be expressed presynaptically in this layer (see Fig. 2B). The additional occurrence of Kv11.2 in dense packages in the IPL indicates the expression of Kv11.2 subunits presynaptically in ribbon synapses of both, photoreceptors and bipolar cells. As these synapses are glutamatergic, we performed double-labeling experiments with Kv11.2- and vGluT1-specific antibodies. These experiments revealed an almost perfect co-localization of the Kv11.2 and the vGluT1 immunostaining (Fig. 4A), though the immunostaining for Kv11.2 in the IPL was more diffuse than that for vGluT1. This may either be caused by a membrane-specific expression of Kv11.2 subunits in contrast to the cytosolic expression of vGluT1 or this may point to an additional expression of Kv11.2 subunits in amacrine cells. Additional double-labeling experiments for Kv11.2 together with synaptophysin or GAD2/GAD65 antibodies (Fig. 4B, C) demonstrated that not all presynaptic structures in the IPL exhibited Kv11.2 immunoreactivity and gave no indication for Kv11.2 expression in GABAergic synapses of amacrine cells.

We were not able to perform direct double-labeling experiments with Kv11.1 and Kv11.2 specific antibodies as both antibodies were raised in rabbits. But we had a strong overlap of Kv11.1 and PKCα on the one hand (Fig. 3A) and of Kv11.2 and vGluT1 expression on the other hand (Fig. 4A). In order to elucidate a possible overlap of Kv11.1 and Kv11.2 expression in the retina, we performed co-labeling experiments with Kv11.1 and vGluT1 and with Kv11.2 and PKCα, respectively (Fig. 3C and 4D). Although both assays revealed a strong co-localization in parts of the IPL, Kv11.1 and Kv11.2 subunits showed a different distribution in the IPL. While Kv11.2 immunoreactivity was evenly distributed, the Kv11.1 subunits showed a more stratified expression. Double-labeling experiments with ChAT-specific antibodies revealed that the strata with less Kv11.1 staining were identical to the cholinergic bands of the IPL (Fig. 3D).

**Figure 5:**
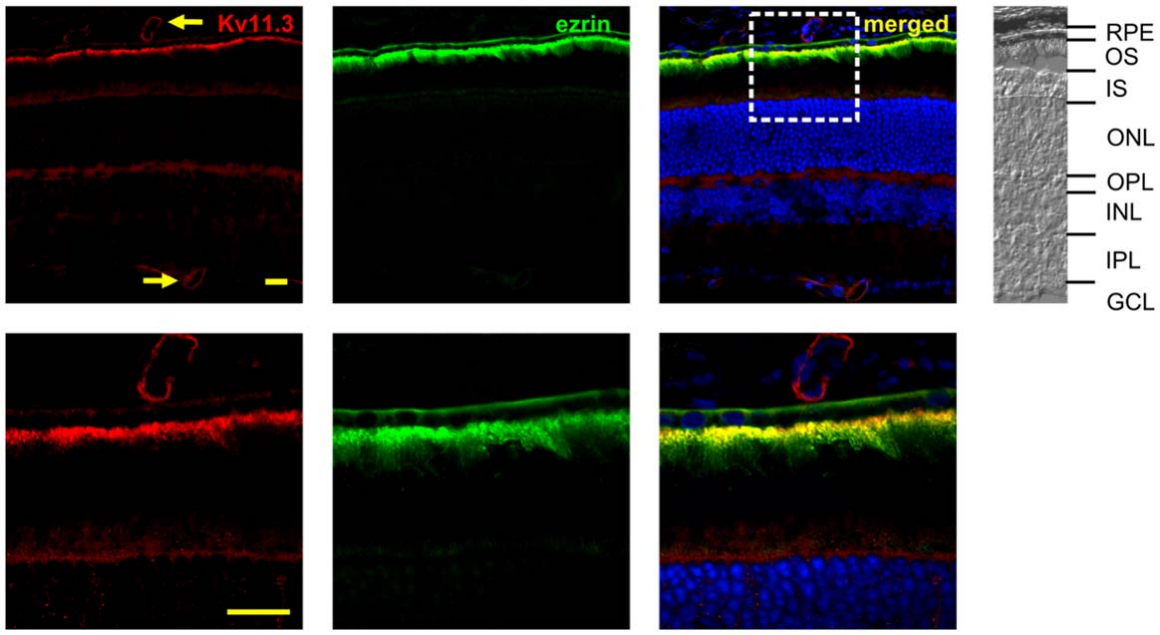
Kv11.3 channel subunits are expressed in the RPE. Ezrin, a marker of the apical membrane of the RPE was used in double-labeling experiments with Kv11.3. The merged data show an almost perfect co-localization of both proteins. Arrows point to the additional expression of Kv11.3 subunits in retinal and choroidal blood vessels. The box highlights the section shown in the bottom row in higher magnification illustrating the coexpression of K11.3 and ezrin in the apical membrane of the RPE. A part of the corresponding bright field picture is shown on the right. RPE: retinal pigment epithelium; OS: outer segments; IS: inner segments; ONL: outer nuclear layer; OPL: outer plexiform layer; INL: inner nuclear layer; IPL: inner plexiform layer; GCL: ganglion cell layer. Scale bars: 20 µm. The results of counter staining of cell nuclei (blue) is included in the merged pictures shown on the right.

Although immunostaining with Kv11.3-specific antibody revealed no immunoreactivity in the neuronal retina, there was a strong signal in the apical membrane of the retinal pigment epithelium (RPE). Double-labeling experiments with an antibody against ezrin, a marker of the apical membrane of the RPE confirmed the expression of Kv11.3 subunits in the apical membrane of the RPE (Fig. 5). Additional immunoreactivity was detected in blood vessels of the retina and choroid (arrows in Fig. 5). This very constricted expression of Kv11.3 channel subunits explains the low levels of Kv11.3 RNA we and others detected in the retina (Fig. 1C and [7]).

## Discussion

The present PCR data show for the first time the mRNA expression of all three Kv11 channel subunits in the human retina and we confirmed and further analyzed their expression in the mouse eye. To unveil the distinct cellular expression patterns of the three Kv11 channel subunits we performed immunohistological studies. While Kv11.1 subunits displayed a relatively wide-spread distribution in different retinal layers, Kv11.2 subunit expression was detected predominantly presynaptically in the glutamatergic photoreceptors and bipolar cells, and Kv11.3 immunoreactivity was detected in the apical membrane of the retina supporting cell layer of the RPE and in choroidal and retinal blood vessels. The RPE plays a major role in spatial buffering of ions in the subretinal space [15]. In the dark, the cGMP-gated inward current of Na^+^ and Ca^2+^ into the outer segments of photoreceptors is counterbalanced by a K^+^ outward current in the inner segments, the so called dark current [16]. Upon illumination the K^+^ current in the inner segment becomes smaller and the K^+^ concentration in the subretinal space decreases from 5 to 2 mM. This is compensated by an increased K^+^ outward current in the apical membrane of the RPE through Kir7.1 inward rectifier channels [17]. But also in the dark most K^+^ ions that enter the RPE through the apical Na^+^/K^+^/2 Cl^-^-ATPase is recycled to the subretinal space [15]. Kv11.3 channels with their relatively negative activation threshold compared to Kv11.1 and Kv11.2 [7], [18], are well suited to contribute to this recycling of K^+^ ions to the subretinal space in the dark and thereby to the maintenance of the resting membrane potential in the RPE which lies around -45 mV.

Interestingly, Kv11.1 displays a distribution in the mouse retina comparable to that of Kv10.1 (eag1) [6]. Like Kv10.1, Kv11.1 was found in IS of photoreceptors, in the OPL, the INL and the IPL. But in contrast to Kv10.1, Kv11.1 was not found in the ganglion cell layer and in the INL it is mainly expressed in the outer part where horizontal and rod bipolar cells dominate. As we could not detect any Kv11.1-specific immunoreactivity in cells positive for the horizontal cell marker calbindin, we exclude prominent Kv11.1 subunit expression at least in the somata of these cells. The co-localization of Kv11.1 with PKCα in the part of the IPL next to the GCL of the mouse retina demonstrates that Kv11.1 channel subunits are also expressed in axon terminals of rod bipolar cells. In both cell types, in photoreceptors and in rod bipolar cells, cGMP plays a dominant role in light-evoked changes of the membrane potential. In photoreceptors cGMP is cleaved upon illumination leading to the closure of cGMP-gated cation (CNG) channels in the outer segments. Even though it is known that cGMP also essentially contributes to light-evoked signal transduction in rod bipolar cells, its transduction pathway in these cells is only partly understood [19]. Despite the existence of a nucleotide binding site in all Kv11 subunits, a modulation of Kv11 channels by rises in cGMP levels has only recently been shown for the Kv11.1b splice-variant and heteromultimeric Kv11.1a/1b channels, whereas it is missing in homomeric Kv11.1a channels. Kv11.1 current inhibition was found to depend on the activation of cGMP-dependent kinase (cGK or protein kinase G) [11]. Since we detected comparable levels of the two splice-variants 1a and 1b in the retina by our real-time PCR experiments, it is tempting to speculate that these subunits also form cGMP/cGK-sensitive heteromultimeric Kv11.1 channels in the retina. In photoreceptors, we detected Kv11.1 channels in the inner segments where they may support the depolarization driven by the CNG channels as these Kv11.1 channels would be inhibited in the dark when the CNG channels are open and vice versa in the light. In rod bipolar cells the rod signal is inverted so that illumination leads to a depolarization. This depolarization is mediated by the metabotropic glutamate receptor mGluR6. In the dark, mGluR6 activation leads to the closure of a the cation channel TRPM1 and thus to the hyperpolarization of the rod bipolar cell [20]. In ON bipolar cells, cGMP has been shown to shift the threshold for detectable events so that dim light flashes become detectable in the presence of cGMP [19]. This effect of cGMP also required cGK activation. As Kv11.1b and Kv11.1a/1b channels are inhibited by cGMP/cGK, these channels could well contribute to the regulation of the response sensitivity of bipolar cells by reducing the hyperpolarizing counter current in the presence of cGMP.

Other modulations of retinal Kv11 channels which might be involved in adaption could include metabotropic GluR1-mediated Kv11 current inhibition as recently shown in mitral cells [21] or changes in the biophysical properties of the three Kv11 channels by altered extracellular K^+^ or Ca^2+^ concentrations [22], [23].

Kv11.2 channels are expressed presynaptically in glutamatergic synapses of the retina, suggesting their contribution to the synaptic release of glutamate in photoreceptors and bipolar cells. The axons of OFF bipolar cells terminate in the outer half of the IPL, whereas those of ON and rod bipolar cells terminate in the inner half of the IPL [2]. Accordingly, the co-localization of Kv11.2 and vGluT1 throughout the IPL strongly suggests that Kv11.2 subunits are expressed in all types of bipolar cells, but not in amacrine cells which is supported by the absence of Kv11.2 and GAD65 co-staining. The double-labeling experiments with PKCα or vGluT1 and Kv11.1 or Kv11.2 have shown that the expression patterns of Kv11.1 and Kv11.2 subunits may overlap only in the axon terminals of bipolar cells in the IPL. This suggests that a formation of heteromeric Kv11.1/2 channels is only possible in this area of the retina. Nevertheless, in contrast to the Kv11.2 immunoreactivity which was evenly distributed throughout the IPL, the Kv11.1 staining was strongest in the rod bipolar cell axon terminals and exhibited a slightly stratified distribution in the more distal parts of the IPL. The two strata with reduced Kv11.1 immunoreactivity represent the location of the dendrites of cholinergic amacrine cells (Fig. 3D). Given that nitric oxide (NO) is an activator of the cGMP/cGK pathway, it might be noteworthy that NO synthase immunoreactivity in the IPL has been found to be enriched in three bands [14] which roughly coincide with the stratification of Kv11.1 staining observed in the present study.

Taken together, our immunohistological data revealed very different expression patterns for the three Kv11 channel subunits in the retina. These differences favor distinct functional roles for the channels in the retina. Kv11.1 channels, possibly heteromers formed by 1a and 1b subunits, may be involved in the setting of the excitability of rod bipolar cells and photoreceptors. In contrast, Kv11.2 channels with their presynaptic expression could be involved in the fine tuning of the presynaptic membrane potential thereby adjusting the neurotransmitter release in these cells. In sharp contrast to the expression of Kv11.1 and Kv11.2 in the neuronal retina, Kv11.3 immunoreactivity was found to be expressed in the RPE where the channels could contribute to the spatial buffering of K^+^ ions.

## Supporting Information

Figure S1 The Kv11 antibodies used for the immunostaining of retina slices were tested for their suitability using heterologously expressed Kv11 channels. HEK cells were transiently transfected with rat cDNA for Kv11.1, Kv11.2 and Kv11.3 tagged with EGFP. Bright field pictures are shown in the left panels. Kv11 channel expression directly indicated by the EGFP fluorescence (middle panels) coincided with Kv11 channel immunoreactivity visualized by the red fluorescent secondary antibody (right panels). Although both, the Kv11.1 antibody and the Kv11.2 antibody showed some cross-reactivity with Kv11.2 and with Kv11.1 channels, respectively, this cross-reactivity was obviously too weak for immunohistology because both channels showed completely different expression patterns (e.g. in the OPL. Compare Fig. 3C and Fig. 4A). Working dilutions of the primary erg channel antibodies were 1∶5000 (AB5930), 1∶5000 (CR1) and 1∶5000(TB2).
(TIF)
