# Supplementary figures and images for: Expression Pattern of Kv11 (Ether à-go-go-Related Gene; erg) K+ Channels in the Mouse Retina
Source: PLoS One. 2011 Dec 19;6(12):e29490. doi: 10.1371/journal.pone.0029490 (PMC3242786; doi:10.1371/journal.pone.0029490)

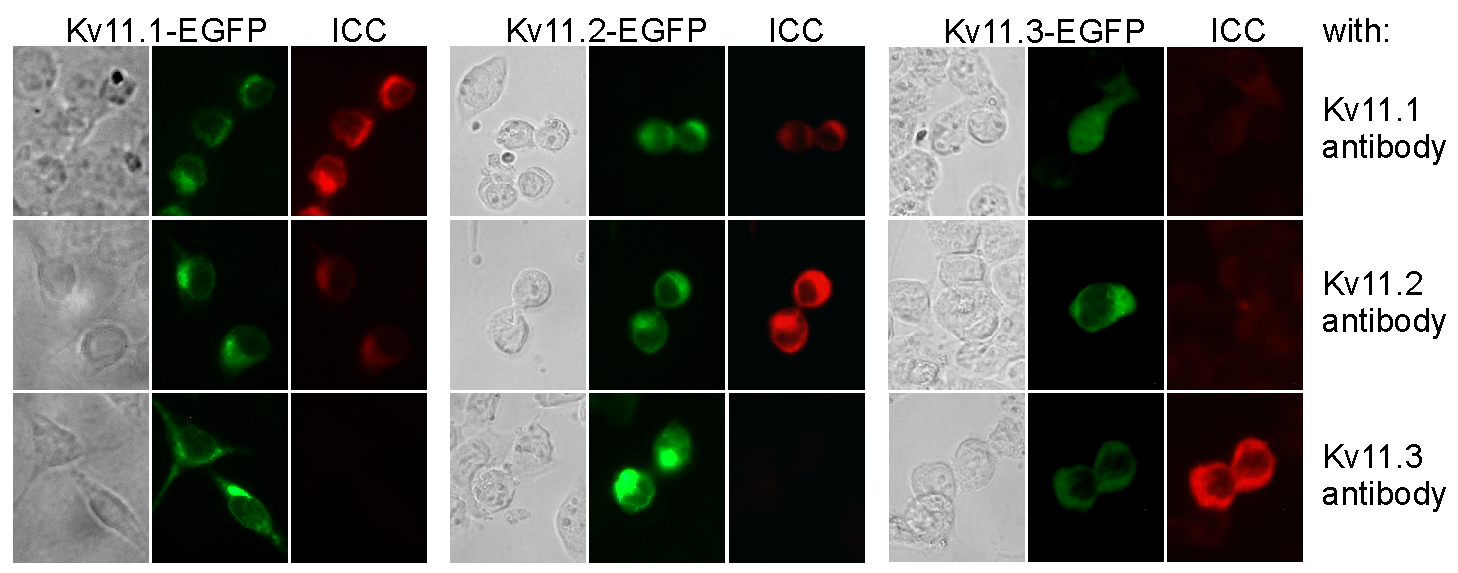

Supplement: Figure S1 — The Kv11 antibodies used for the immunostaining of retina slices were tested for their suitability using heterologously expressed Kv11 channels. HEK cells were transiently transfected with rat cDNA for Kv11.1, Kv11.2 and Kv11.3 tagged with EGFP. Bright field pictures are shown in the left panels. Kv11 channel expression directly indicated by the EGFP fluorescence (middle panels) coincided with Kv11 channel immunoreactivity visualized by the red fluorescent secondary antibody (right panels). Although both, the Kv11.1 antibody and the Kv11.2 antibody showed some cross-reactivity with Kv11.2 and with Kv11.1 channels, respectively, this cross-reactivity was obviously too weak for immunohistology because both channels showed completely different expression patterns (e.g. in the OPL. Compare Fig. 3C and Fig. 4A). Working dilutions of the primary erg channel antibodies were 1∶5000 (AB5930), 1∶5000 (CR1) and 1∶5000(TB2). (TIF) [file pone.0029490.s001.tif]
